# GWAS Reveals a Novel Candidate Gene *CmoAP2/ERF* in Pumpkin (*Cucurbita moschata*) Involved in Resistance to Powdery Mildew

**DOI:** 10.3390/ijms23126524

**Published:** 2022-06-10

**Authors:** Hemasundar Alavilli, Jeong-Jin Lee, Chae-Rin You, Yugandhar Poli, Hyeon-Jai Kim, Ajay Jain, Kihwan Song

**Affiliations:** 1 Department of Bioresources Engineering, Sejong University, Seoul 05006, Korea; alavilli.sundar@gmail.com (H.A.); jjstar9@naver.com (J.-J.L.); chaerytree@naver.com (C.-R.Y.); kimhj8897@naver.com (H.-J.K.); 2 ICAR-Indian Institute of Rice Research, Hyderabad 500030, India; poliyugandhar@gmail.com; 3 Amity Institute of Biotechnology, Amity University Rajasthan, Jaipur 303002, India; ajain2@jpr.amity.edu

**Keywords:** pumpkin, *Cucurbita moschata* Duchesne ex Poir., *Podosphaera xanthii*, powdery mildew, *CmoAP2/ERF*, genome-wide association study, single nucleotide polymorphism, powdery mildew resistance

## Abstract

Pumpkin (*Cucurbita moschata* Duchesne ex Poir.) is a multipurpose cash crop rich in antioxidants, minerals, and vitamins; the seeds are also a good source of quality oils. However, pumpkin is susceptible to the fungus *Podosphaera xanthii*, an obligate biotrophic pathogen, which usually causes powdery mildew (PM) on both sides of the leaves and reduces photosynthesis. The fruits of infected plants are often smaller than usual and unpalatable. This study identified a novel gene that involves PM resistance in pumpkins through a genome-wide association study (GWAS). The allelic variation identified in the *CmoCh3G009850* gene encoding for AP2-like ethylene-responsive transcription factor (*CmoAP2/ERF*) was proven to be involved in PM resistance. Validation of the GWAS data revealed six single nucleotide polymorphism (SNP) variations in the *CmoAP2/ERF* coding sequence between the resistant (IT 274039 [PMR]) and the susceptible (IT 278592 [PMS]). A polymorphic marker (dCAPS) was developed based on the allelic diversity to differentiate these two haplotypes. Genetic analysis in the segregating population derived from PMS and PMR parents provided evidence for an incomplete dominant gene-mediated PM resistance. Further, the qRT-PCR assay validated the elevated expression of *CmoAP2/ERF* during PM infection in the PMR compared with PMS. These results highlighted the pivotal role of *CmoAP2/ERF* in conferring resistance to PM and identifies it as a valuable molecular entity for breeding resistant pumpkin cultivars.

## 1. Introduction

The Cucurbitaceae family comprises~120 genera and more than 800 species distributed in the tropical and subtropical regions of the world and includes several economically important fruit and vegetable crops, such as cucumber, melon, pumpkin, squash, and watermelon [1]. The genus *Cucurbita* contains several wild-type species and five major species domesticated globally as cash crops, i.e., *Cucurbita argyrosperma* K. Koch, *Cucurbita ficifolia* Bouché, *Cucurbita maxima* Duchesne, *Cucurbita moschata* Duchesne ex Poir., and *Cucurbita pepo* L. [2]. *C. moschata* Duchesne ex Poir. (pumpkin; 2*n* = 40) is closely related to *C. argyrosperma* K. Koch and is the second most diverse species in the genus after *C. pepo* L. The pumpkin fruit and seeds exhibit extensive morphological diversity [3]. Pumpkin is an herbaceous climbing or creeping vine that grows well in warm tropical areas and is cultivated and consumed across the globe in the form of mature and immature young stems, tendrils, flowers, fruits, and seeds [3,4]. Pumpkins provide a good source of nutrients, including amino acids, carbohydrates, carotenoids, flavonoids, phenolics, and provitamin A (β-carotene and α-carotene), and the seeds are a good source for oil extraction, which has several therapeutic properties [5,6,7,8]. Pumpkins are also widely used as a grafting rootstock for other cucurbit crops [3]. Pumpkins rootstocks strengthen biotic and abiotic stress tolerance in cucurbit crops and are used as a bloomless rootstock for cucumbers to produce a glossy green fruit, which enhances consumer favoritism and marketability [9,10].

Powdery mildew (PM) disease is caused by different species of obligate biotrophic plant pathogenic fungi in the order Erysiphales, which produce conidia profusely and infect a wide range of crops, adversely affecting their growth and yield potential [11]. The PM infection can be easily detected by its characteristic whitish, powdery mycelial growth and conidia formation on the adaxial and abaxial surfaces of the leaves, vines, petioles, stems, flowers, and fruits [12,13]. *Podosphaera xanthii* is one of the predominant hard-to-control fungi causing PM on all the species of the Cucurbitaceae family, including cucumber, melon, pumpkin, squash, and watermelon [11,14,15,16]. *P. xanthii* is prevalent in most countries, including Korea [16]. *P. xanthii* uses haustoria for the acquisition of nutrients from the host tissue [17]. *P. xanthii* mycelium can overwinter in the buds of infected plants, and its spores spread over great distances through air, irrigation water, and soil, which makes the mitigation of PM an arduous process [15,17,18]. Fluctuating high humidity and temperature, along with dry weather, further aggravate the transmission of PM by *P. xanthii* [15,17,18,19]. Since cucurbits are grown in high humidity and warm conditions, they are highly susceptible to *P. xanthii* during the blossom and developing stage of the young fruits, and their leaves are most susceptible [18]. *P. xanthii* infection in cucurbits triggers chlorosis and early desiccation and senescence of the leaves; it also affects the flavor, quality, and marketability of the fruits due to the development of sunscald [13,15]. Fungicides are conventionally used for managing cucurbit PM infection, but high incidence of fungicide resistance of *P. xanthii* obstructs disease management and is a concern for human health and the environment [15,16,20,21]. Therefore, there is an urgent need for developing cucurbits resistant to *P. xanthii* using other alternative approaches, e.g., the application of epiphytic fungus [22], bacterial culture by-products [23], and natural products and oils [24], or through the breeding of resistant varieties [13,25]. Relatively, molecular breeding is the most accurate, cost-effective, and sustainable approach for breeding pumpkins resistant to *P. xanthii* compared to the time-consuming and labor-intensive conventional approach.

In 2000, *Arabidopsis thaliana* was the first plant genome sequenced by using a Sanger-based bacterial artificial chromosome (BAC)-to-BAC approach [26]. Since then, second-generation sequencing technologies, e.g., Illumina and Roche 45, spurred an exponential increase in the high-quality sequencing of plant reference genomes [27,28,29]. In 2009, *Cucumis sativus* L. was the first cucurbit to be sequenced [30]. However, it was after almost a decade, in 2017, that a high-quality genome of *C. moschata* was sequenced and assembled, comprising a total of 80.1 Gb of high-quality cleaned Illumina paired-end and mate-pair generated reads representing 215× coverage of its genome and an estimated (based on 17-mer depth distribution) genome size of 372 Mb [31]. However, functional genomics is a difficult task for sequenced *C. moschata*. Genome-wide association studies (GWAS) have efficaciously mapped thousands of loci associated with complex horticultural traits, including PM resistance in cucumber germplasm [32,33,34]. However, there have been no studies on GWAS of PM in *C. moschata.*

In this study, we screened 407 pumpkin germplasms from the global diversity panel and evaluated their responses to PM for identifying PMR and PMS pumpkin cultivars at the early seedling and adult stages. GWAS, in conjunction with phenotyping and the genotyping by sequencing (GBS) derived SNP, led to the identification of the *CmoAP2/ERF* gene. The genotyping marker was developed based on the allelic diversity of the *CmoAP2/ERF* in PMR and PMS pumpkin lines. Further, augmented relative expression of *CmoAP2/ERF* in PMR suggested its potential role in conferring tolerance to PM. Thus, the study revealed the potency of allelic variations in *CmoAP2/ERF* for the early and efficient detection of PM resistance in pumpkin cultivars.

## 2. Results

### 2.1. Screening for PM-Resistant Pumpkin Germplasm

**Figure 1:**
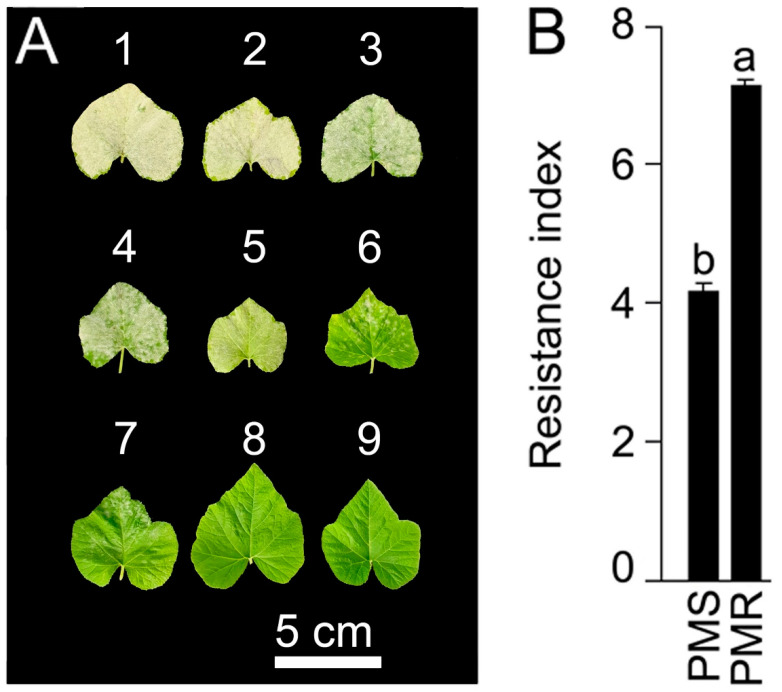
Visual scoring of PM resistance. At an experimental farm, 407 pumpkin genotypes were grown to first true leaf stage (~14 d after germination). Seedlings were infected twice at 5 d intervals, with PM suspensions collected from the heavily infected plants. After PM infection, leaf samples were gently removed, and the resistance index was scored on a scale of 1–9. (**A**) 1–4: susceptible; 5 and 6: moderately resistant; 7–9: resistant. (**B**) The PMS and PMR were compared for their resistance index. Values are means ± SE (*n* = 4), and different letters on the histograms indicate that the values differ significantly (*p* < 0.05).

From the global diversity panel, 407 pumpkin accessions were collected and evaluated for their response to simulated PM infection at the fully expanded first true leaf stage (~14 d after germination) consecutively for two years (2018–2019) at the experimental farm of Sejong University. The resistance index was scored as susceptible (1–4), moderately resistant (5 and 6), and resistant (7–9) (Figure 1A). Based on the resistance index in consecutive screenings, the pumpkin accessions IT 274039 and IT 278592 were consistently identified as powdery mildew resistant (PMR) and powdery mildew susceptible (PMS), respectively (Figure 1B). PMS and PMR accessions were subsequently used for generating the F_2_ population to identify the potential gene responsible for conferring resistance to PM.

### 2.2. Multivariate GWAS Analysis for PM Resistance in Pumpkin

**Figure 2:**
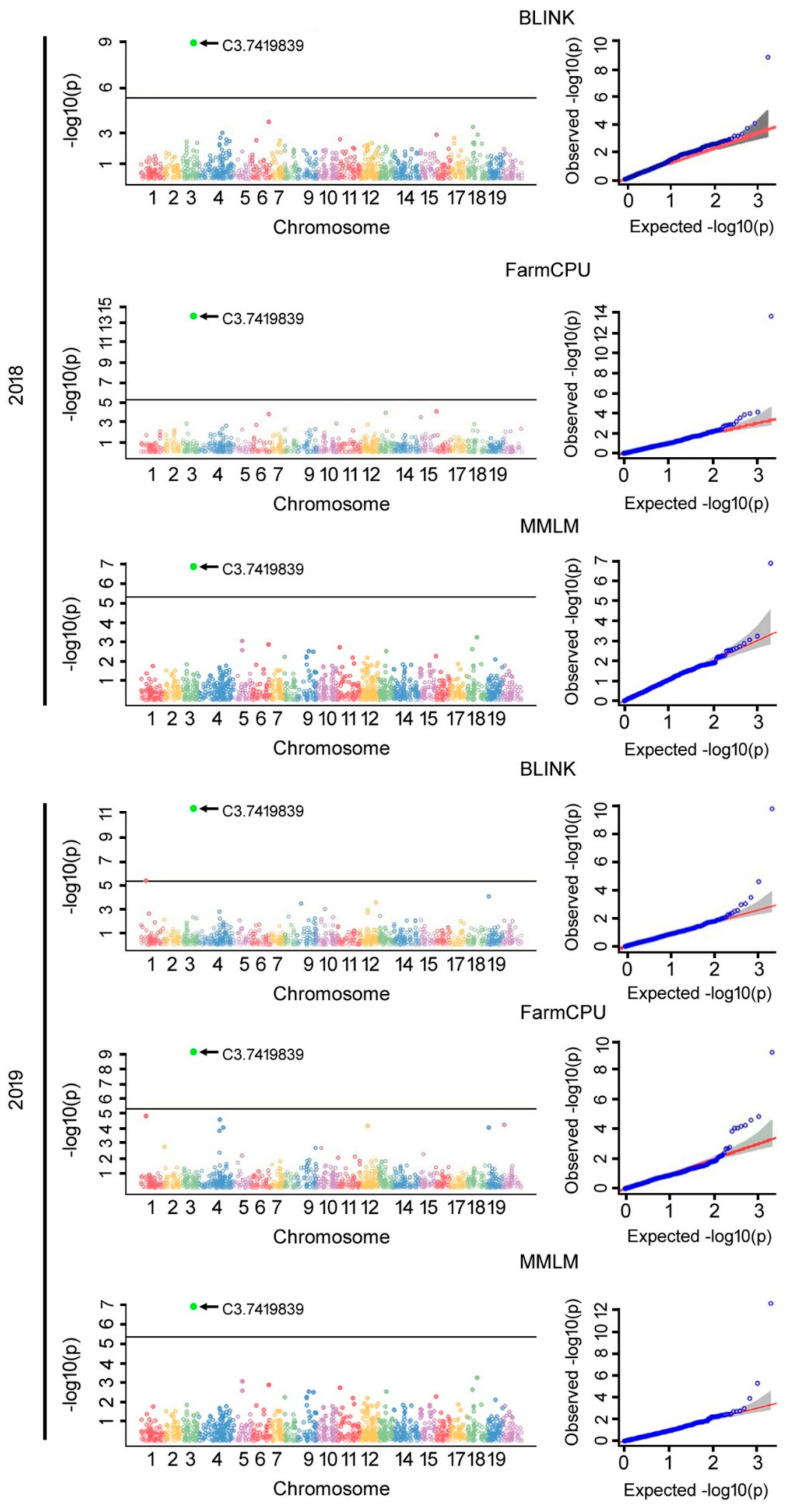
Multivariate GWAS models BLINK, FarmCPU, and MMLM predicted PM resistance in pumpkins. Over a two-year study (2018 and 2019), different multi-locus based GWAS models, i.e., BLINK, FarmCPU, and MMLM, were used for the prediction of potential SNPs associated with PM resistance in 407 pumpkin genotypes. The black arrow denotes most significant association. In the Manhattan plot, the horizontal solid line indicates the Bonferroni-corrected significance threshold value at –log10(*p*) of 4.61. Different colors in the Manhattan plots represents different chromosomes in pumpkin. For different models, the Manhattan and quantile–quantile plots are presented on the left and right panels, respectively.

Phenotypic data on resistance to PM were collected from 407 pumpkin accessions over a period of 2 years (2018–2019). Further, GBS analysis led to the identification of 2071 high-quality SNPs distributed across 20 chromosomes of pumpkins [3]. Using this data, we used the Genome Association and Prediction Integrated Tool (GAPIT) program for GWAS analysis. The GAPIT is a single R-language based platform that combines the most powerful and computationally efficient GWAS models [35]. The GAPIT package comprises several multi-locus, e.g., Bayesian-information and Linkage-disequilibrium Iteratively Nested keyway (BLINK) [36], Fixed and Random Model Circulating Probability Unification (FarmCPU) [37], and Multi Locus Mixed Model (MLMM) [38] and single locus, e.g., General Linear Model (GLM) [39], Mixed Linear Model (MLM) [40], and Settlement of MLM Under a Progressively Exclusive Relationship (SUPER) [41] GWAS models. In our study, a threshold of Bonferroni-corrected significance at a –log10(*p*) value of 4.61 was determined (Figure 2 and Table 1). Initially, the MLM model data predicted only 2 SNPs (C3. 7419839 and C3. 7419891) (Table 1 and Supplementary Figure S1). In addition to these two SNPs, the SUPER model predicted 3 more SNPs (C3. 7078924, C15. 7755972, and C15. 7756005) (Table 1 and Supplementary Figure S1). In addition to the 5 SNPs predicted by the SUPER model, another SNP (C3. 8060138) was predicted by the GLM model (Table 1 and Supplementary Figure S1). The models, such as BLINK, FarmCPU, and MLMM, were employed to further reduce misleading positives (Figure 2 and Table 1). All three multi locus models predicted only one SNP, C3. 7419839 that was positioned at chromosome 3 (Figure 2). Hence, it is thought to be the most reliable SNP. Furthermore, the GLM, SUPER, and MLM models also commonly predicted one more SNP at C3. 7419891, which is 52 nucleotides distant from C3. 7419839 (Figure 2 and Supplementary Figure S1). Together, we selected C3. 7419839 and C3. 7419891 as the significant SNPs to investigate further.

### 2.3. Candidate Gene Identification 

**Figure 3:**
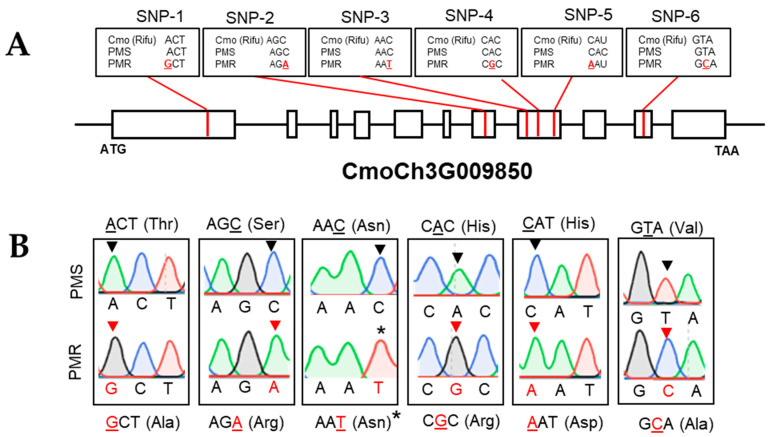
Candidate gene identification and allelic diversity assessment. (**A**) The gene structure of the *CmoAP2/ERF* gene and a map of the location of the missense mutations in the exon region. (**B**) A comparison of the PMS and PMR line Sanger-sequencing chromatograms and the resultant amino acid changes. The inverted black triangle in the upper panel indicates the PMS line or reference genome, whereas the inverted red triangle in the lower panel indicates the nucleotide changes in the PMR line. The asterisk indicates the SNP variation, but no amino acid change.

To validate the novel SNPs identified in the GWAS analysis, we compared their physical location with the previously reported major pumpkin QTL associated with the PM resistance in chromosome 3 [42]. The SNPs, including C3.7419839 and C3.7419891 as the primary candidates predicted from the multivariate GWAS analysis, were positioned approximately 1.42 Kb away from the previously reported major QTL region (~400 kb region between C3. 7562022~C3. 7981972 nucleotides) in chromosome 3 [42]. Further, sequence analysis revealed that the C3. 7419839 and C3. 7419891 SNPs were indeed present in the 8th exon of *CmoCh3G009850* gene encoding for *APETALA2 (AP2)*-like ethylene-responsive transcription factor (*CmoAP2/ERF)* (Figure 3A). The *CmoAP2/ERF* gene contains a polypeptide of 491 amino acid (AA) residues, with an estimated molecular weight of 54.3 KDa and an isoelectric point of 5.81, as predicted by a web-based protein analysis tool (www.bioinformatics.org/sms/prot_mw.html (accessed on 8 February 2022)). The computational analysis predicted the presence of two conserved domains in the *CmoAP2/ERF* protein, such as an AP2 superfamily domain spanning from AA151 to AA212 residues and an AP2 domain spanning from AA243 to AA306 (www.ncbi.nlm.nih.gov/Structure/cdd/wrpsb.cgi accessed on 8 February 2022) (Supplementary Figure S2).

### 2.4. Validation of SNPs and Allelic Diversity Analysis

To substantiate the SNPs indicated by the GWAS analysis, we employed direct gene sequencing for the *CmoAP2/ERF* gene using the gDNA extracted from the PMS and PMR parents. The sequencing data asserted the presence of 4 more point mutations and the 2 SNPs predicted in the GWAS analysis in the PMR line *AP2-ERF* gene coding sequences (Figure 3B). Among the 6 SNPs, C3. 7418367 (SNP-1) is located in exon-1, C3. 7419693 (SNP-2) is found in exon-7, C3. 7419839 (SNP-3), C3. 7419850 (SNP-4), and C3. 7419891 (SNP-5) are located in exon-8, and C3.7420273 (SNP-6) is found in exon-10 (Figure 3A). Among the two GWAS-predicted SNPs, one of the SNP at C3. 7419839 (SNP-3) is found to be a synonymous mutation, and it does not cause any amino acid change, despite the nucleotide change (Figure 3A,B). However, the other SNP at C3. 7419891 (SNP-5) is a nonsynonymous mutation, and it resulted in an amino acid change at AA 321 (Figure 3A,B). Likewise, all the remaining four SNPs (SNP 1, 2, 4, and 6) were nonsynonymous mutations and resulted in substituting different amino acid residues at AA105, AA302, AA321, AA402, respectively (Figure 3A,B). In line with these findings, many earlier reports substantiated the roles of the AP2/ERF transcription factor family genes for mediating the pathogen responses in diverse plant species [43,44,45].

### 2.5. Genotyping Marker Development and Segregation Analysis for PM Resistance 

**Figure 4:**
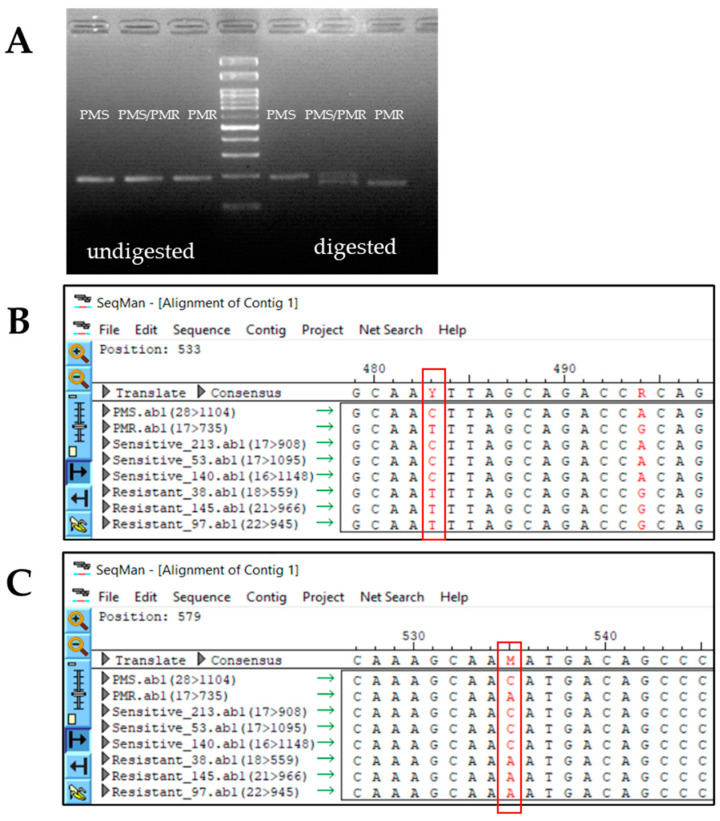
Profiles of designed SNPs, differentiating PMS and PMR lines. (**A**) Amplification profile of pumpkin lines showing polymorphism of the *CmoAP2/ERF* dCAPS marker. The PMS, PMS/PMR, and PMR lines amplified using the *CmoAP2/ERF* dCAPS marker (**right**) and band patterns after restriction digestion with *Pst1* (**left**). (**B**) Coding sequence alignment of PMS/PMR lines, along with three PM sensitive and three PM resistant lines for SNP verification. Sequence alignments of selected lines and the red box indicate SNP variation at SNP C3. 7419839. (**C**) Coding sequence alignment of PMS/PMR lines, along with three PM sensitive and three PM resistant lines, for SNP verification. Sequence alignments of selected lines and the red box indicate SNP variation at SNP C3. 7419891.

Using the CAPS and dCAPS approach, we designed a genotyping marker using the GWAS-predicted SNPs at positions C3. 7419839 and C3. 7419891. However, we could not find suitable polymorphic markers at this position to distinguish the PMS and PMR parents (data not shown). As the gene sequencing result revealed a few more SNP variations between the PMS and PMR, we used one of the SNPs (C3.7420273) at exon 10 for marker development. For validating this dCAPS marker, we initially used both parental lines (PMS and PMR) and the pooled parental line (PMS + PMR) gDNA as a heterogenous control. We confirmed that the designed marker conspicuously differentiates the controls (Figure 4A).

Previously, by visual assessment, we sorted some clear PM sensitive and resistant lines from the PMS/PMR-F_2_ progeny. The phenotyping data based on our analysis obtained 86 susceptible, 120 moderately resistant, and 39 resistant lines, giving a ratio of 1:1.39:0.45 (Supplementary Table S1). Later, we quantified the diseased leaf area (DLA) using Image J software, and genotyped them using the dCAPS marker, developed based on the SNP variation in C3. 7420273. The phenotyping and genotyping results clearly agreed with each other (Supplementary Table S2). Further, we were interested to learn whether the sensitive and resistant lines (identified by visual observation/ImageJ/genotyping) comprise the GWAS-predicted SNP variations. For this purpose, we selected three susceptible and three resistant lines (confirmed by three approaches) and sequenced them, along with the PMS and PMR parental controls (Figure 4B,C). Intriguingly, the susceptible and resistant lines contain the GWAS-predicted SNP variation, and the genotyping marker can be used for F_2_ segregation analysis. Further, we used the same marker to genotype 238 PMS/PMR-F_2_ population. We assumed a segregation ratio of 1:2:1, as per single-gene Mendelian inheritance, in the F_2_ genotyping. On the contrary, our genotyping analysis revealed 80 sensitive, 109 heterozygotes, and 49 resistant lines, giving a ratio of 1:1.36:0.61 (Table 2). Based on the phenotypic and genotypic data, the PM-resistant gene shows an incomplete dominant inheritance, so the segregation ratio is distorted from the Mendelian single-gene inheritance. We speculate that the segregation deviation might be due to the lower fitness of the resistant genes, introgressed into the *C. moschata* genome through an interspecific hybridization (wide crossing) event from the wild inedible gourd *C. martinezii*.

### 2.6. Scoring and Sorting the PMS/PMR-F_2_ Lines Based on the Genotyping Result

**Figure 5:**
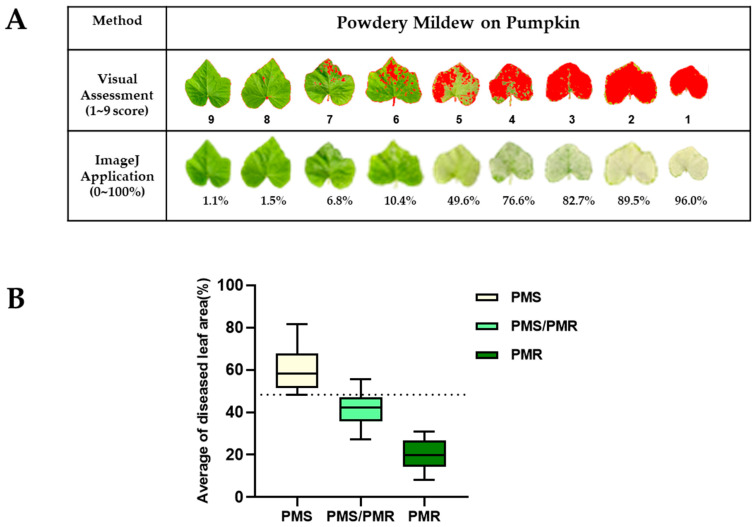
Quantification and comparison of diseased leaf area by visual assessment and Image J. (**A**) The performance of the visual assessments and ImageJ for the same leaves of various disease severities of powdery mildew. The red color implies disease severity whereas the white color means tolerance (**B**) Assortment of ImageJ quantified PM infected PMS/PMR-F_2_ population based on the genotyping results. The box plots depict the diseased leaf area (DAL) percentage, and the dotted line indicates the threshold of resistance.

The quantification of disease symptoms is critical for evaluating disease severity, and it is a prerequisite for making the best disease management decisions [46]. Based on the visual assessment, we initially selected PM sensitive, moderate resistant, and resistant lines on a scale of 1–9. Further, we imaged each line for quantitative data, measured the DLA using the ImageJ program, and matched their intensity scale (Figure 5A). After genotyping all the PMS/PMR-F_2_ population with the *CmoAP2/ERF* dCAPS marker, we verified what percentage of Image J quantified DLA value can be used as a threshold for consideration as susceptible, moderately resistant, or resistant. In our analysis, the homozygous susceptible lines held with a DLA percentage range of 48.3–81.6%, whereas the moderate resistant or heterozygote lines ranged between 38.9–55%. On the other hand, the homozygous resistant lines had DLA values between 8.02–30.75%, respectively (Figure 5B). These results imply that the DLA values correlate with the *CmoAP2/ERF* gene zygosity, and at least the *CmoAP2/ERF* homozygous resistant lines can effectively resist PM infestation.

### 2.7. Expression Changes of CmoAP2/ERF Gene in PMR Line

**Figure 6:**
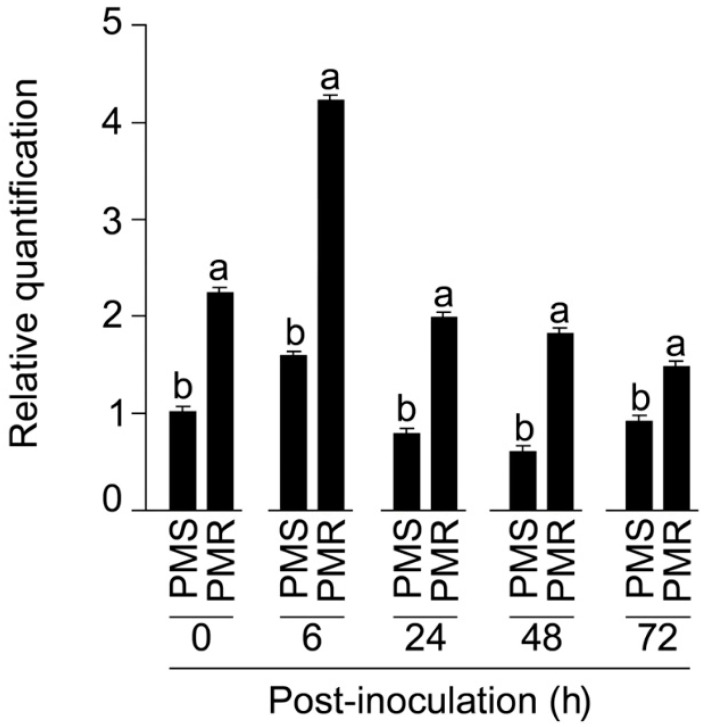
Expression of the *CmoAP2/ERF* gene in PMS and PMR lines at different time intervals post-PM infection. Relative expression of *CmoAP2/ERF* gene in PMS and PMR lines in response to PM pathogen infection. The *CmoActin* gene was used as an internal control for normalization. For each experiment, the expression level of the PMS sample prior to pathogen treatment was used as a calibrator for quantification and was assumed as 1. Values are means ± SE (*n* = 3), and different letters on the bars indicate that the values differ significantly (*p* < 0.05).

To investigate whether the endogenous expression of *CmoAP2/ERF* expression correlates with different post-infection intervals, we performed qRT-PCR and assessed the *CmoAP2/ERF* transcript levels in the PMS and PMR lines. In the real-time experiment, we found that endogenous *CmoAP2/ERF* expression is already (before the pathogen treatment) significantly higher in the PMR line compared to the PMS line (Figure 6). Moreover, the *CmoAP2/ERF* transcript augmentation peaked after 6 h post-infection and started to decline from 24 h to 72 h post-infection in the PMR line. Nevertheless, *AP2/ERF* expression remained higher in the PMR line than in the PMS line at all the examined time points. (Figure 6). Therefore, the higher endogenous expression of *CmoAP2/ERF* in the PMR lines might plausibly be involved in quick defense response and enhanced the expression of downstream powdery mildew-resistant genes.

## 3. Discussion

Being sessile-natured, the plants have innately evolved with intricate signaling pathways against biotic stress. The transcription factors (TFs) are the key players in the plant defense circuit to fight against various pathogens [47]. Many TF families have thus far been identified for their response to bacterial, fungal, and viral infections [47,48]. Our study uncovered a novel allelic variation in the *CmoAP2/ERF* transcription factor and demonstrated that augmented endogenous expression of the *CmoAP2/ERF* gene might improve the resistance in PMR lines.

In pursuing resistance breeding, identifying PM-resistant germplasm resources is critical for the breeding of resistant varieties. Specifically, many breeders have resorted to the wild crop progenitors. These bestow rich genetic diversity and great potential to fortify the modern cultivars with either biotic or abiotic stress tolerance [49,50]. Previously, for breeding of PM resistant varieties, many wild cultivars were identified in various crops, including barley [51], wine grape [52,53], strawberry [54], and wheat [44]. Likewise, in the case of cucurbits, the wild progenitors, including *C. okeechobeensis* subsp. *martinezii* and *C. lundelliana* were found with PM-resistant traits and extensively used for cultivar development [42]. Through interspecific hybridization, a major powdery mildew resistance region from the wild species *C. okeechobeensis* subsp. *martinezii* was introgressed into *C. pepo* and *C. moscahata,* [55,56,57]. In line with these findings, True French, a commercial *C. pepo* cultivar, was crossed with a PM resistant wild cultivar, *C. okeechobeensis* subsp. *Martinezii,* and the resistance gene was described as *Pm-0* [56,57].

In our study, the phenotyping results of the PMS/PMR-F_2_ revealed a ratio of susceptible: partially resistant: resistant as 1:1.39:0.45, which suggests incomplete dominant resistance (Supplementary Table S1). Accordingly, the *CmoAP2/ERF* dCAPS marker genotyping revealed a ratio of susceptible: heterozygous: resistant as 1:1.36:0.61. This implies a lower fitness of the resistant gene, likely due to its introgression into the *C. moschata* genome via distant hybridization or wide crossing. Although the trait inheritance is incomplete, the heterozygotic lines were found to exhibit adequate PM resistance. This phenomenon agrees with the previous report on the interspecific hybrid between *C. pepo* and *C. martinezii* [56,58]. Nevertheless, these seemingly contradicting Mendelian ratios are not uncommon. For example, few qualitative PM-resistance related studies in wheat, including *pmX* [59] and *pmWE99* [60], and two stem rust-resistant genes, including *Sr36* [61] and *Sr40* [62], also showed segregation distortion patterns due to the favored transmission of specific alleles. Another possible explanation for segregation distortion in our study could be the functional association of the *CmoAP2-ERF* gene in floral organs, reproductive, and post-embryonic tissue development processes (cucurbitgenomics.org/feature/gene/CmoCh03G009850 (accessed on 25 April 2022)). Interestingly, we also found that the homozygous PMR lines that harbor the incomplete dominant resistance suffered a negative growth impact on their seed production greater than that of the PMS lines (Supplementary Figure S3), which might also, at least in part, contribute to the segregation distortion.

Since the first report on *C. moschata* whole-genome sequencing was published in 2017 [31], the utilization of pumpkin genomic resources was made available for trait discovery and marker-assisted breeding for crop improvement applications [42]. Particularly while utilizing the next-generation sequencing tools, the GBS strategy aided in high-throughput genotyping and the identification of several genome-wide SNP markers in multiple crop species [63]. In a recent report, Park et al. (2020) carried out GBS analysis and SNP mapping in pumpkins leading to the identification of a single major QTL region in chromosome 3 (~spanned in a 400 kb region between C3. 7562022~C3. 7981972) associated with powdery mildew resistance [42]. In line with these findings, Lee et al. (2020) analyzed 610 pumpkin germplasms by utilizing the GBS approach and reported 2071 high-quality SNPs distributed across the 20 pumpkin chromosomes [3]. A few other recent studies also elucidated some of the *C. moschata* genes associated with the PM responses through transcriptome profiling [4]. In their study, Guo et al. identified that six pumpkin genes, including *bHLH87* (basic helix–loop–helix 87), *ERF104* (ethylene response factor 104), *WRKY21*, *HSFA* (heat shock factor A), *MLO3* (mildew locus O), and *SGT1* (suppressor of G2 allele of SKP1) were differentially regulated in PM-resistant line compared to the PM-sensitive line [4]. Moreover, the *CmoSGT1* and *CmobHLH87* genes were ectopically overexpressed in tobacco and found to enhance the PM resistance in the transgenic lines [64,65].

While GWAS is a potent technique for dissecting complex traits associated with biotic and/or abiotic stresses in crops, which helps in identifying the novel candidate genes and/or loci [66], one of the challenges is to maintain a stringent check on the false positives generated by the family relatedness and population structure [67]. Therefore, several single-locus and multi-locus based GWAS models were required to evade the false negatives. The GAPIT is a single composite suite which comprises of most powerful and computationally efficient GWAS models [35]. In our study, while using the GAPIT suite, we processed our data through both multi-locus (BLINK, FarmCPU, and MMLM) and single locus (GLM, MLM, and SUPER) GWAS models (Table 1). Two novel SNPs, such as C3.7419839 and C3.7419891, were commonly predicted by single-locus based models (Supplementary Figure S1), whereas the multi-locus models predicted only one SNP, C3. 7419839 (Figure 2). Apparently, these two SNPs were positioned in exon 8 of the *CmoAP2/ERF* gene in chromosome-3 (Figure 3A). Further, Sanger sequencing affirmed both the GWAS-predicted SNPs and additionally uncovered 4 more SNP variations among the PMS and PMR haplotypes (Figure 3A). Further, the qRT-PCR analysis substantiated the higher endogenous *CmoAP2/ERF* expression prior to pathogen treatment, and this is also found to be augmented in response to PM infection (Figure 6). Therefore, we suggest that the *CmoAP2/ERF* is one of the key regulator genes, and that it is highly associated with the pumpkin PM responses. Additionally, we validated a dCAPS marker that could specifically detect the *CmoAP2/ERF* gene zygosity (Figure 4A), which could be beneficial for marker-assisted breeding and stacking the *CmoAP2/ERF* gene with other PM-resistant genes.

The AP2/ERF group of transcription factors is the largest group of plant-specific TFs. This group is widely known for its multifarious roles, including plant development and biotic and abiotic stress regulation [1,43,44]. A recent report revealed that the *C. moschata* genome contains 212 AP2/ERF genes [68]. In the past, several ERF genes were characterized in the model land plant *Arabidopsis thaliana* for their functional association with plant pathogenic responses against *Alternaria alternata*, *Botyosphaeria dothidea*, *Pseudomonas syringae* PV tomato DC3000 (PstDC3000), *Ralstonia solanacearum*, and tobacco mosaic virus (TMV) [43]. Likewise, the *ERF* genes from one species were functionally characterized in other plant species for their roles in enhancing tolerance to pathogen attack. For instance, overexpression of *Malus domestica ERF 100* (MdERF100) in *A. thaliana* [43]. *Gossypium hirsutum* ERF gene (GhB301) in *Nicotiana benthamiana* [69,70], *Artemisia annua ORA* (AaORA) gene in *A. thaliana* [71], *Glycine max ERF5* (GmERF5) in soybean [45], and tobacco stress-induced gene 1 (Tsi1) in hot pepper [72] enhanced transgenic line tolerance to different pathogens. Furthermore, several reports ascertained the *ERF* gene functional association with both biotic and abiotic stress-responsive gene elements [44,73,74]. Hence, we speculate the *CmoAP2/ERF* gene expression might also be instrumental in conveying abiotic stress tolerance as well. Disrupting the function of disease-sensitive genes is also an appealing approach for mitigating PM-induced damage [75]. Using technological advances, such as CRISPR technology, a number of recent studies have shown that knocking down PM sensitive gene expression can effectively render disease resistance in field crops, such as tomato [75], grape [76], sweet basil [77], and wheat [78], without any growth penalty. The next step will be to look at the functional characterization of the *CmoAP2/ERF* gene in other commercially important crop plants to see if it can help with abiotic stress tolerance, as well as PM resistance.

## 4. Materials and Methods

### 4.1. Plant Materials and Growth Conditions

A total of 407 pumpkin accessions were obtained from the National Agrobiodiversity Centre, Rural Development Administration (RDA) in Korea and from the Agricultural Research Service (ARS) of the United States Department of Agriculture (USDA); the accessions were advanced through self-pollination for 3 cycles. The entire collection was planted in plastic houses at the experimental farm facility at Anseong-si, Republic of Korea, for two consecutive years between, 2018~2019. Further, we used them for the evaluation of powdery mildew (PM) disease responsive phenotypes when the seedlings were in the fully expanded first true leaf stage. Two replications from three plants of each accession were examined throughout the growing period, as described in our previous report [3].

### 4.2. Disease Evaluation and Scoring of PM Response

Healthy pumpkin seedlings at the fully expanded first true leaf stage were used to evaluate the PM response. The conidial suspensions were collected from the heavily diseased plants for inoculation. The density of the spore suspension was adjusted to approximately 1 × 10^5^ sporangia per ml before the inoculation. The inoculation was repeated 2 times in 5 day intervals. The resistance index was scored 2 times (10 and 14 days) after the final inoculation day. The disease severity and categorization were briefly estimated using the resistance scale of 1–9, where 1–4: susceptible, 5–6: moderate resistance, 7–9: resistance.

For quantification of the diseased leaf area (DLA), or the percentage of the F_2_ progeny, we employed Image J (www.imagej.nih.gov/ij (accessed on 13 January 2022)) online software. Briefly, image of each infected pumpkin leaf sample was captured using a Samsung Galaxy S105G equipped with 12 mega pixel rear camera, 6 mm wide, and F2.4 (telephoto). To achieve uniformity, all the image background was removed using a web-based program called remove.bg (www.remove.bg accessed on 13 January 2022). Further, we imported the processed images to ImageJ and applied the same color threshold value for all the samples to obtain accurate scoring. The infection density was calculated using the formula shown below.

DLA (%) = (Damaged area/whole leaf area) × 100

### 4.3. Genome-Wide Association Study

The GAPIT R package [35] was used to perform genome-wide association studies using a dataset of 2071 high-quality SNPs and phenotype data previously generated using 407 examples of pumpkin accessions [3]. Multivariate GWAS methods were employed to evaluate the trait-SNP associations for PM resistance. The significance of associations among markers and traits was given as *p*-value for significant markers (*p* ≤ 0.05). The GWAS result was presented using Manhattan plots [−log10(*p*)] and quantile–quantile (QQ) plots. Based on the Bonferroni test, the threshold value was estimated at a significant level of 5% [−log10 (2.41) = 4.61].

### 4.4. Genomic DNA Extraction and Identification of Candidate Gene

Genomic DNA (gDNA) was extracted using the fresh and turgid pumpkin leaf samples. For the gDNA extraction, we used the cetyltrimethylammonium bromide (CTAB) method [79]. The gDNA quality and integrity was checked by agarose gel electrophoresis, and the quantity was assessed using a Nanodrop ND-2000 spectrophotometer, following the manufacturer′s instructions (Thermo Fisher Scientific, Waltham, MA, USA). In this study, we used *C. moschata* (Rifu) genome (http://cucurbitgenomics.org/organism/9 (accessed on 12 December 2021)) sequences as the pumpkin reference sequences for comparison. To ascertain the in silico SNP data and to affirm the allelic diversity, we sequenced the *CmoAP2/ERF* gene from both the variants. For this purpose, we extracted gDNA from the PMS and PMR lines, and PCR was amplified using the gene specific primers. Further, we checked the amplification, followed by the purification of the PCR fragments using a gel purification kit (Cosmo Genetech, Seoul, Korea) and submitted the samples for sequencing (Cosmo Genetech, Seoul Korea) using the gene specific primers.

### 4.5. Development of Genotyping Markers and Validation

Based on Sanger gene sequencing, we found many single nucleotide polymorphic (SNP) variations between the PMS and PMR lines. We used one of the SNPs for developing the genotyping markers by using the web-based tool called dCAPS finder (http://helix.wustl.edu/dcaps/dcaps.html (accessed on 1 February 2022)). Based on the program output, we examined various primers and identified one of the dCAPS markers that can distinguish the PMS and PMR lines. The primer pairs used for PCR amplifications are *CmoAP2/ERF*-dCAPS-for- 5′ CAATTCAGGCGGCAGGCGGGTGCTG 3′ and *CmoAP2/ERF-* dCAPS- rev-5′ ATTATT CGGCCTCCA TTACT 3′. Upon PCR with the specified primers, followed by the *Pst1* (New England Biolabs, Ipswich, MA, USA) enzyme digestion, the amplicons were resolved in 5% agarose gel.

### 4.6. RNA Isolation and cDNA Synthesis

For the gene expression analysis, the PMR and PMS lines were grown in plastic pots in a greenhouse facility at Sejong university, Seoul, South Korea. The *p. xanthii* inoculum was prepared from the heavily infected leaves and sprayed evenly on the seedling surface. The tissues were harvested from the PMS and PMR samples at 0, 6, 24, 48, and 72 h, respectively. The samples were snap frozen in liquid nitrogen and kept at −80 °C for RNA isolation. The total RNAs were isolated from the pumpkin leaf samples using the TaKaRa MiniBEST Plant RNA Extraction Kit (Shiga, Japan). First-strand cDNAs were synthesized using the Nanohelix Easy cDNA synthesis kit (Daejon, Korea).

### 4.7. Quantitative Real Time PCR (qRT PCR)

qRT-PCR was performed using Toyobo Thunderbird SYBR qPCR mix (Osaka, Japan) and carried out in CFX96 connect Real-time Quantitative system (Bio-Rad Laboratories, Hercules, CA, USA). The relative expression of *CmoAP2/ERF* (CmoCh03G009850) gene expression in different samples was calculated by normalizing the expression values with those of the housekeeping gene *CmoActin* (CmoCh11G015080) as an endogenous control. For calculating the relative gene expression of the *CmoAP2/ERF* gene, we adopted the 2^–∆∆Ct^ method, as previously described [80,81]. The relative expression of the *CmoAP2/ERF* gene was estimated using the primer pairs *CmoAP2/ERF*-qRT-For 5′ GAACGACTGCCCTGATGTGA 3′ and *CmoAP2/ERF*-qRT-Rev 5′ AAGCCGATCCCACCCTTTTC 3′. As an endogenous control, we used *CmoActin*-qRT-For- 5′ CCGCTCTTGCTCCGAGCAG 3′ and *CmoActin*-qRT-Rev- 5′ ATCCACATCTGTTGGAAGGTAC 3′, respectively. Three independent biological replicates for each treatment were prepared. For each biological replicate, we ran at least three technical replicates of each PCR reaction.

### 4.8. Bioinformatic Analysis

To visualize the Sanger gene sequencing-derived chromatograms and sequence analysis, a web-based program, snap gene viewer suite (www.snapgene.com/snapgene-viewer (accessed on 1 March 2022)), was used. For assembling and comparing the Sanger gene sequencing data, we used DNASTAR Seqman analysis software. For designing primers, the Primer 3 web-based tool was used (https://primer3.ut.ee (accessed on 15 February 2022)).

### 4.9. Statistical Analysis

All statistical comparisons between the variances were determined by Student′s *t*-test and least significant differences (LSD) using Statistix 8.1 computation software. The chi-square test was performed using Microsoft Excel 16.0 software to estimate the segregation ratio of PM resistance in the F_2_ progeny derived from a cross between the PMS and PMR lines.

## 5. Conclusions

Pumpkins are economically important crops used as food stuffs and rootstocks for cucurbits worldwide. However, PM caused by *p.xanthii* affects the growth of pumpkins and reduces plant productivity. GWAS is a viable strategy for associating specific genetic variations with a particular trait. The use of GWAS for the resistance to PM in pumpkin resulted in the identification of a novel gene encoding *CmoAP2/ERF,* with six SNP variations in the coding sequence of the haplotypes PMR and PMS. Further qRT-PCR exhibited an elevated expression of *CmoAP2/ERF* during PM infection in PMR compared with PMS. Overall, the study provided empirical evidence for a pivotal role of the genotyping marker developed based on the allelic variations in *CmoAP2/ERF* for rapidly detecting *p.xanthii*-mediated PM resistance in pumpkin genotypes.

## Figures and Tables

**Table 1:** Overview of highly significant marker associations for PM resistance identified using multivariate GWAS methods.

Year	Model	Marker	Chromosome	Position	*p*-Value	−log10 Value
**2018**	BLINK	C3. 7419839	3	7419839	1.22 × 10^−9^	8.9135
	FarmCPU	C3. 7419839	3	7419839	2.13 × 10^−14^	13.6708
	GLM	C3. 7419839	3	7419839	3.72 × 10^−9^	8.4290
		C3. 7419891	3	7419891	7.16 × 10^−7^	6.1449
		C3. 8060138	3	8060138	7.25 × 10^−7^	6.1394
		C3. 7078924	3	7078924	1.64 × 10^−6^	5.7857
		C15. 7755972	15	7755972	2.74 × 10^−6^	5.5625
		C15. 7756005	15	7756005	2.74 × 10^−6^	5.5625
	MLMM	C3. 7419839	3	7419839	1.30 × 10^−7^	6.8876
	MLM	C3. 7419839	3	7419839	4.17 × 10^−7^	6.3799
		C3. 7419891	3	7419891	7.73 × 10^−6^	5.1118
	SUPER	C3. 7419839	3	7419839	1.07 × 10^−9^	8.9688
		C3. 7419891	3	7419891	1.72 × 10^−7^	6.7632
		C3. 7078924	3	7078924	1.11 × 10^−6^	5.9539
		C15. 7755972	15	7755972	6.83 × 10^−6^	5.1655
		C15. 7756005	15	7756005	6.83 × 10^−6^	5.1655
**2019**	BLINK	C3. 7419839	3	7419839	4.60 × 10^−12^	11.3373
	FarmCPU	C3. 7419839	3	7419839	7.29 × 10^−10^	9.1372
	GLM	C3. 7419839	3	7419839	5.88 × 10^−11^	10.2306
		C3. 7419891	3	7419891	2.75 × 10^−10^	9.5609
		C3. 8060138	3	8060138	2.10 × 10^−7^	6.6785
		C3. 7078924	3	7078924	8.96 × 10^−7^	6.0478
		C15. 7755972	15	7755972	7.39 × 10^−6^	5.1311
		C15. 7756005	15	7756005	7.39 × 10^−6^	5.1311
	MLMM	C3. 7419839	3	7419839	2.58 × 10^−13^	12.5876
	MLM	C3. 7419839	3	7419839	5.49 × 10^−9^	8.2605
		C3. 7419891	3	7419891	7.82 × 10^−9^	8.1066
	SUPER	C3. 7419839	3	7419839	6.88 × 10^−11^	10.1621
		C3. 7419891	3	7419891	3.01 × 10^−10^	9.5218
		C3. 7078924	3	7078924	7.89 × 10^−7^	6.1030
		C15. 7755972	15	7755972	7.32 × 10^−6^	5.1355
		C15. 7756005	15	7756005	7.32 × 10^−6^	5.1355

**Table 2:** Co-segregation analysis of the *CmoAP2/ERF* gene in the PMS x PMR-F2 population.

Cross	Number of F_2_ Plants	PMS	PMS/PMR	PMR	Ratio	χ^2^	*p*-Value
PMS/PMR	238	80	109	49	1:1.36:0.61	9.41	3.84

## Data Availability

Data is available in the manuscript and in the Supplementary Materials.

## Supplementary Materials

The following supporting information can be downloaded at: www.mdpi.com/article/10.3390/ijms23126524/s1.
