# Supplementary material for: GWAS Reveals a Novel Candidate Gene CmoAP2/ERF in Pumpkin (Cucurbita moschata) Involved in Resistance to Powdery Mildew
Source: Int J Mol Sci. 2022 Jun 10;23(12):6524. doi: 10.3390/ijms23126524 (PMC9223685; doi:10.3390/ijms23126524)
Supplement: Supplementary file 1 [file ijms-23-06524-s001.zip › ijms-1745753-supplementary.pdf]

Supplementary Table S1. Co-segregation analysis of PM resistance phenotype.

Generation	Total Numbers of Plants/Families	Visual observation			Expected ratio	Observed ratio	$\chi 2$	*p*-value
		Susceptible	Moderately resistant	Resistant				
		(1-4)	(5-6)	(7-9)				
PMS	18	18						
PMR	18			18				
F2	245	86	120	39	1:2:1	1: 1.39: 0.45	13.33	3.84

PMS and PMR indicate a susceptible parent and resistant, respectively.

Supplementary Table S2. Validation of GWAS predicted SNP variation in the selective susceptible and resistant PMS x PMR F2 lines.

PMS x PMR-F2 plant number	Visual assessment	Image J (DLA%)	Genotype	Phenotype
140	2.5	81.2	Sensitive	Sensitive
213	2.75	73.4	Sensitive	Sensitive
53	3	73.2	Sensitive	Sensitive
38	8	0.27	Resistant	Resistant
97	7	8.02	Resistant	Resistant
145	7	10	Resistant	Resistant

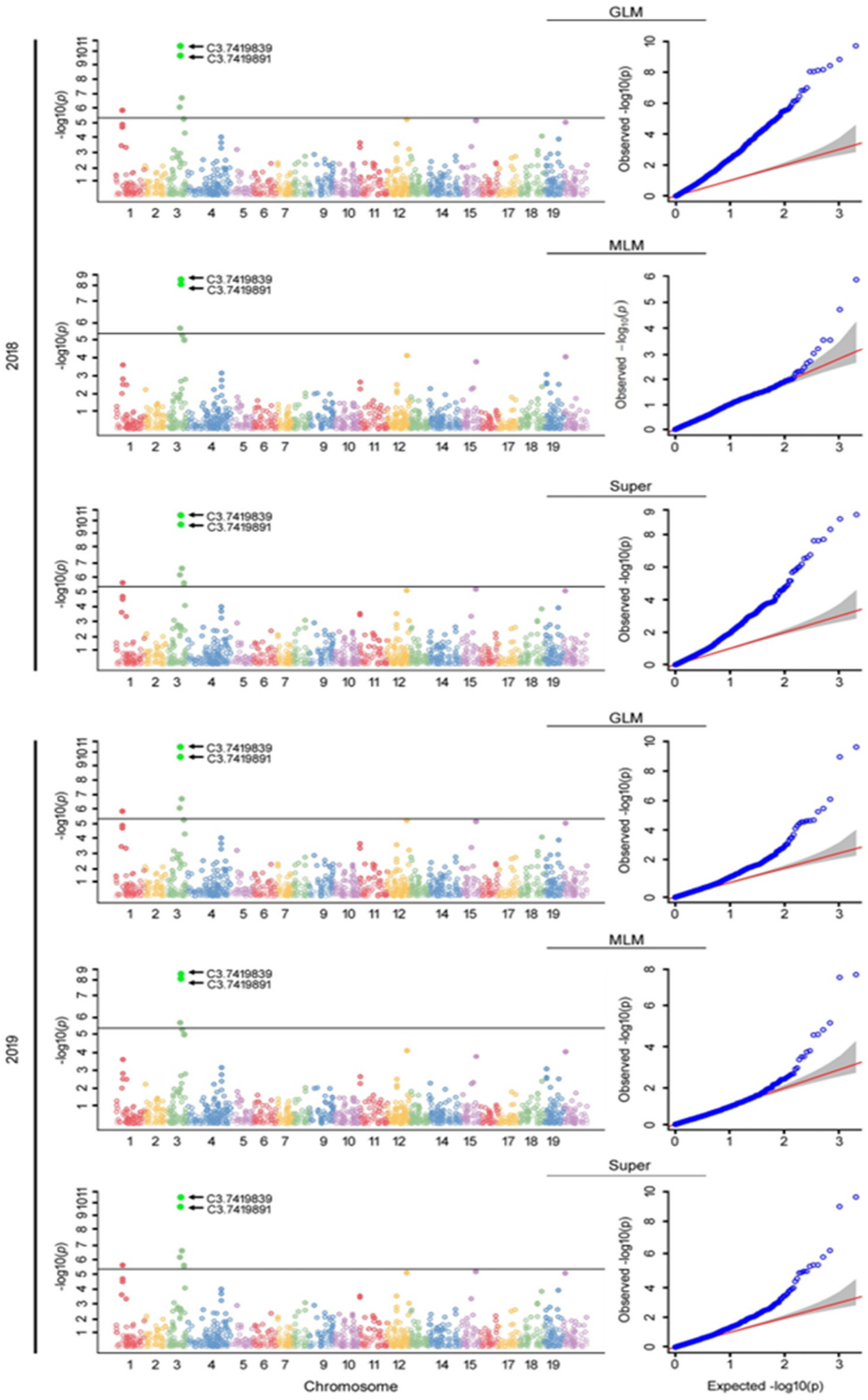

Supplementary Figure S1. Multivariate GWAS models GLM, MLM & CMLM, and SUPER predicted PM resistance in pumpkins. Over a two-year study (2018 and 2019), different single locus based GWAS models, i.e., GLM, MLM & CMLM, and SUPER were used for the prediction of potential SNPs associated with PM resistance in 407 pumpkin genotypes. Black arrow denotes most significant association. In the Manhattan plot, the horizontal solid line indicates the Bonferroni-corrected significance threshold value at –log10(*P*) of 4.61. For different models, Manhattan and Quantile-Quantile plots are presented on the left and right panels, respectively.

MWDLNDCPDVREGDESEACSSAKTSIEGDGDDDKRKRVGSASNSSCSAAEEEEELEEEEEEEEGSDEVLGSMVMQID

TQTPSPVTRQFFPLEDTEIPPPLPLPTTDSAFPRAHWVGVKFGHPDPNNNSINPNDLSHPIKKSRRGPRSRSSQYRG

VTFYRRTGRWESHIWDCGKQVYLGGFDTAHAAARAYDRAAIKFRGVEADINFSIEDYEDDLKQMGNLTKEEFVHVLR

APETALA2 superfamily domain

*

RQSTGFPRGSSKYRGVTLHKCGRWEARMGQFLGKKYVYLGLFDTEIEAARAYDKAAIKCNGKEAVTNFDPSIYESEL

APETALA2 domain

NPTTDSTSNLADHSLDLSLGNSSSKQHDSPNGSLDPHHSSSAPLPMEADWQRSQGFRPPQLNLLQESSSGGGSGNKN

YNNAQRGNTNRRQAGGVDQPHSQILLHNQNHPTFNYQIQFPGSSNGGRIIGSDLSLSLSDHQNQVVESGPSQVHATA

AASSGFSPQIRPSKNWLHKNGFHCLIRPS

Supplementary Figure S2. Deduced amino acid sequence of CmoAP2/ERF protein was retrieved from NCBI. The yellow colour highlight denotes APETALA2 super family domain, and the pink colour highlight denotes APETALA2 domain. The Asterisk in APETALA2 domain indicates the position of a missense mutation occurred in PMR line.

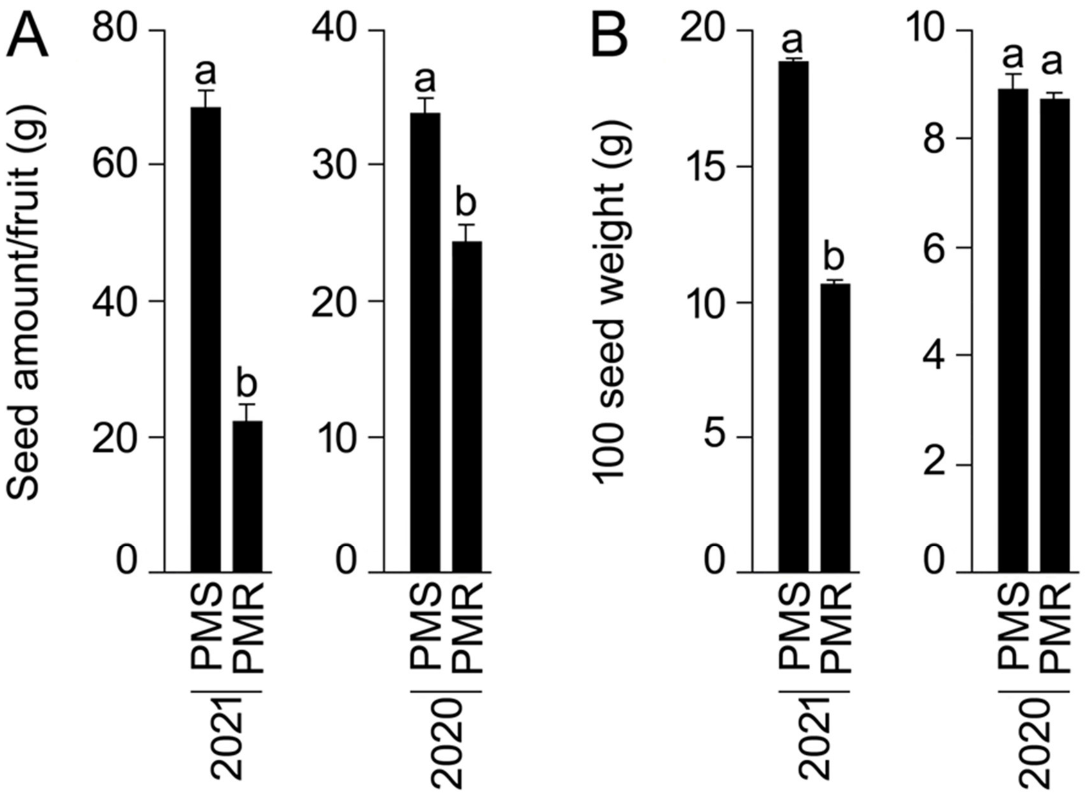

Supplementary Figure S3. Comparison of seed yield-related traits between PMS and PMR. (**A**). Amount of seed weight/ fruit in 2018 and 2019 (**B**). Seed weight/100 number of seeds in 2018 and 2019. Values are means ± SE (n = 4) and different letters on the histograms indicate that the values differ significantly ($p < 0.05$).
